# Adipose Tissue Dysfunction in Nascent Metabolic Syndrome

**DOI:** 10.1155/2013/393192

**Published:** 2013-04-04

**Authors:** Andrew A. Bremer, Ishwarlal Jialal

**Affiliations:** ^1^Department of Pediatrics, Vanderbilt University, Nashville, TN 37232-9170, USA; ^2^Laboratory for Atherosclerosis and Metabolic Research, University of California Davis Medical Center, Sacramento, CA 95817-2218, USA; ^3^VA Medical Center, Mather, CA 95655-4200, USA

## Abstract

The metabolic syndrome (MetS) confers an increased risk for both type 2 diabetes mellitus (T2DM) and cardiovascular disease (CVD). Moreover, studies on adipose tissue biology in nascent MetS uncomplicated by T2DM and/or CVD are scanty. Recently, we demonstrated that adipose tissue dysregulation and aberrant adipokine secretion contribute towards the syndrome's low-grade chronic proinflammatory state and insulin resistance. Specifically, we have made the novel observation that subcutaneous adipose tissue (SAT) in subjects with nascent MetS has increased macrophage recruitment with cardinal crown-like structures. We have also shown that subjects with nascent MetS have increased the levels of SAT-secreted adipokines (IL-1, IL-6, IL-8, leptin, RBP-4, CRP, SAA, PAI-1, MCP-1, and chemerin) and plasma adipokines (IL-1, IL-6, leptin, RBP-4, CRP, SAA, and chemerin), as well as decreased levels of plasma adiponectin and both plasma and SAT omentin-1. The majority of these abnormalities persisted following correction for increased adiposity. Our data, as well as data from other investigators, thus, highlight the importance of subcutaneous adipose tissue dysfunction in subjects with MetS and its contribution to the proinflammatory state and insulin resistance. This adipokine profile may contribute to increased insulin resistance and low-grade inflammation, promoting the increased risk of T2DM and CVD.

## 1. Introduction

The metabolic syndrome (MetS) comprises a cluster of cardiometabolic risk markers with insulin resistance and adiposity as central features [1–4]. Five diagnostic criteria for MetS have been identified (central obesity, dyslipidemia (high triglycerides (TGs) and/or low high-density lipoprotein cholesterol (HDL-C)), hypertension, and impaired fasting glucose) by the Adult Treatment Panel III (ATPIII) criteria of the National Cholesterol and Education Program (NCEP), and the presence of three of these features is considered sufficient to diagnose the syndrome [2, 4, 5]. Using this definition, the National Health and Nutrition Examination Survey (NHANES) data show that currently ~35% of all US adults have MetS [6] and that >40% of adults over the age of 50 have the syndrome [7]. It is important to emphasize that the diagnosis of MetS has been harmonized using the NCEP ATPIII criteria with the exception of different cut-points for waist circumference for different races [8]. Furthermore, MetS confers an increased risk for cardiovascular disease (CVD) and type 2 diabetes mellitus (T2DM) [7, 9–12], both of which are additional risk factors for increased morbidity and mortality.

Numerous investigators have shown increased circulating biomarkers of inflammation in MetS, thus providing support for the syndrome's proinflammatory state [2, 4, 13]. Furthermore adipokine biology has been extensively detailed in recent reviews, and hence it will not be the focus of this paper [14–16]. However, there are scant data on adipose tissue biology in individuals with nascent MetS (a term coined by us to denote subjects with MetS but without the confounding presence of diabetes and/or cardiovascular diseases) [17]. The relationship between inflammation and MetS is supported by several studies [2, 4, 18, 19], as is the relationship between increased visceral fat mass and MetS [20–22]. However, there is a paucity of data on subcutaneous adipose tissue (SAT) biology in the pathogenesis of MetS [23]. 

The subcutaneous fat—which comprises ~80% of adipose tissue and is the major source of fatty acids for the liver—is readily accessible to study and has been shown to be metabolically correlated to indices of insulin resistance as well as to visceral adipose tissue (VAT) [24–27]. In addition to intra-abdominal fat, investigators have shown that the amount of SAT in subjects with MetS positively correlates with increasing MetS factor scores and negatively correlates with circulating adiponectin levels [28]. Other investigators have also reported that SAT is significantly associated with MetS and increases with the increasing number of MetS features, independent of age and sex [29]. Furthermore, inflammatory cells and processes, such as macrophage infiltration, appear to be important in adipose tissue inflammation. Specifically, investigators have examined abdominal SAT from obese subjects and reported that an inflamed adipose phenotype characterized by tissue macrophage accumulation in crown-like structures (CLSs) is associated with systemic hyperinsulinemia and insulin resistance and impaired endothelium-dependent flow-mediated vasodilation [30]. Macrophage retention in fat was also linked to upregulated tissue CD68 and tumor necrosis factor-alpha (TNF-*α*) mRNA expressions in addition to increased plasma high-sensitivity C-reactive protein (hsCRP) concentrations. 

Although it appears that chronic low-grade inflammation could be a central feature to explain the increased risks of CVD and diabetes in MetS, the precise mechanisms remain to be elucidated. As such, our laboratory has focused on the potential role of SAT dysregulation in the syndrome's pathogenesis. Specifically, given the paucity of data examining SAT biology and plasma adipokines in subjects with nascent MetS, we have investigated the role of SAT in MetS and its contribution to the syndrome's systemic low-grade inflammatory process.

## 2. Subcutaneous Adipose Tissue Dysregulation in Nascent MetS

To determine whether SAT biology in subjects with nascent MetS is dysregulated and contributes to the syndrome's systemic low-grade inflammatory process, we studied 65 age- and sex-matched adults [31]. Subjects were classified as having MetS or not using the NCEP ATPIII criteria [5]; those classified as MetS had at least three risk factors to sustain the diagnosis, including central obesity, hypertension, dyslipidemia (low HDL-C and high TGs), impaired fasting glucose, and/or hypertension or on antihypertensive medications. The control subjects needed to have ≤2 features of MetS and not be on blood pressure (BP) medications. Other exclusion criteria for controls were a fasting plasma glucose concentration >100 mg/dL and a fasting TG concentration >200 mg/dL. For both groups, other exclusion criteria were a previous diagnosis of diabetes, clinical atherosclerosis (coronary artery disease (CAD), peripheral vascular disease, CVD, etc.), a TG concentration >400 mg/dL, a hsCRP concentration >10 mg/L, pregnancy, an increased complete blood count (CBC), alcohol consumption >1 oz/day, consumption of n-3 polyunsaturated fatty acids, smoking, hypo- or hyperthyroidism, malabsorption, active wounds, recent surgery, inflammatory or malignant disease, anticoagulant therapy, steroid therapy, the current use of anti-inflammatory drugs, statins and/or other hypolipidemic agents, hypoglycemic agents, angiotensin receptor blockers, oral contraceptives, and antioxidant supplements (in the prior 6 months), postmenopausal women on estrogen replacement therapy, and chronic high intensity exercisers (exercise > 100 min/week). 

Fasting plasma samples were collected from all volunteers after informed consent, and SAT biopsies were obtained from the gluteal area. Biomarkers that were examined included adiponectin, CRP, serum amyloid A (SAA), leptin, plasminogen activator inhibitor-1 (PAI-1), retinol-binding protein-4 (RBP-4), chemokines (monocyte chemotactic protein-1 (MCP-1), and interleukin (IL)-8), as well as cytokines (IL-1, TNF-*α*, IL-8, and IL-6). In addition, SAT samples were stained for CD68 (a macrophage marker) and T-cells (CD3 and CD5) to assess macrophage/T-cell infiltration into the adipose tissue, and the numbers of CLS per high power field (HPF) were counted. There were no significant differences in the ages of the participants and the male: female ratio between the controls and subjects with MetS. Since the percent of African Americans in our cohort was only 9 percent [32], we were unable to undertake any realistic subgroup analyses with respect to race. 

Not surprisingly, the waist circumference (WC), body mass index (BMI), BP, fasting glucose concentrations, non-HDL-cholesterol concentrations, TG concentrations, and the homeostasis model assessment (HOMA) for insulin resistance were higher in the MetS subjects than in controls, whereas HDL-C concentrations were lower. Furthermore, the hsCRP, IL-6, IL-1*β*, leptin, SAA, and RBP-4 concentrations were significantly higher in the MetS subjects than in controls, whereas the adiponectin concentrations were lower.

Table 1 shows the concentrations of several biomarkers released from incubated SAT specimens from the controls and subjects with MetS. Expressed per gram of fat, the levels of secreted leptin, RBP-4, CRP, SAA, PAI-1, and MCP-1 were significantly higher in subjects with MetS than controls. Moreover, the SAT release of IL-1*β*, IL-6, IL-8, and MCP-1, as expressed per mg of protein, was higher in SAT from subjects with MetS than controls.

No lymphocyte populations were observed in any of the SAT specimens from the controls and subjects with MetS using CD3 and CD5 staining. However, there were significantly increased numbers of macrophages infiltrating the SAT of MetS subjects compared to controls as demonstrated by positive CD68 staining. Furthermore, there were significantly increased numbers (~3-fold) of CLS in the SAT specimens from MetS subjects than those from controls (controls: 5 CLS/10 hpf; MetS: 14 CLS/10 hpf; *P* < 0.001). Interestingly, the CLS did not correlate with any proinflammatory mediators, suggesting that they are not classical M1 macrophages [31]. The elucidation of the SAT macrophage phenotype in subjects with MetS is critical to understanding its role in the syndrome's pathogenesis. 

Since the patients with MetS in our study cohort had significantly greater WCs than the controls, all the analytes were also evaluated with WC as a covariate. Importantly, in the adjusted analyses, all the reported differences between the MetS and control groups persisted except for RBP-4 (Table 1). Furthermore, a subgroup analysis revealed higher levels of only leptin and RBP-4 in those subjects with a WC over the median, 42 in. versus under 42 in. (for WC <42 in., leptin was 71 (49–83) ng/ml versus 82 (55–89) ng/ml for WC >42 in.; for WC <42 in., RBP-4 was 25 (11–31) g/mg protein versus 33 (17–37) ng/mg protein for WC >42 in.; *P* < 0.05 compared with WC <42 in.).

Because both insulin resistance and low-grade inflammation are typical features of MetS, we used HOMA and hsCRP as the representative biomarkers for these conditions to evaluate relevant correlations as well. HsCRP correlated positively with HOMA (*r* = 0.39, *P* = 0.03) and adipose tissue MCP-1 (*r* = 0.46, *P* = 0.03) and negatively with adipose tissue adiponectin (*r* = −0.44, *P* = 0.01). There were significant correlations between circulating CRP and SAT CRP (*r* = 0.49, *P* = 0.01), IL-1*β* (*r* = 0.56, *P* = 0.002), and IL-6 (*r* = 0.76, *P* = 0.001) as well. Furthermore, HOMA correlated positively with PAI-1 (*r* = 0.56, *P* = 0.02), RBP-4 (*r* = 0.49, *P* = 0.03), and SAT MCP-1 (*r* = 0.039, *P* = 0.04). 

Given the sparse data on adipose tissue biology in nascent MetS, we recently reported our findings evaluating four additional novel adipokines in this cohort, including chemerin, omentin-1, resistin, and visfatin in both plasma and SAT [17]. Importantly, as noted above, none of the subjects studied had diabetes or any chronic inflammatory diseases nor were any on anti-inflammatory, hypolipidemic, or hypoglycemic drugs. Furthermore, all the subjects had CRP levels <10 mg/L, normal CBCs, fasting glucose concentrations between 100 and 125 mg/dL, and a hemoglobin A1c (HbA1c) <6.5%. Not surprisingly, subjects with MetS were more insulin resistant and had higher levels of hsCRP. It is important to emphasize that in this paper 20 percent of the patients with MetS were not obese. 

The circulating levels of chemerin were significantly increased in the subjects with MetS compared to controls, and this significance persisted (*P* = 0.0005) following adjustment for age and BMI or WC (Table 2). There were also significantly lower levels of plasma omentin-1 in subjects with MetS compared to controls (*P* = 0.004); importantly, the significance persisted when the data were adjusted for BMI or WC (Table 2) (*P* = 0.03). Furthermore, there were higher levels of circulating resistin in subjects with MetS compared to controls; however, these differences did not persist following adjustment for BMI or WC. Plasma visfatin levels were not significantly different between the two groups.

Importantly, there was also a significantly higher release of chemerin from SAT in subjects with MetS which persisted following adjustment for BMI or WC and age (Table 2). In addition, there was a significantly lower secretion of omentin from SAT in subjects with MetS which persisted following adjustment for both age and BMI or WC. However, the secretion of both resistin and visfatin from SAT was not significantly different between the MetS and control groups. Plasma chemerin concentrations correlated significantly (*P* < 0.05) with SAT chemerin (*r* = 0.44), hsCRP (*r* = 0.28), HOMA (*r* = 0.42), TG (*r* = 0.41), systolic BP (*r* = 0.28), omentin (*r* = −0.42), and HDL-C (*r* = −0.37). Moreover, both circulating and SAT omentin-1 levels correlated significantly with each other (*r* = 0.44, *P* < 0.05). Plasma omentin-1 concentrations also correlated significantly with glucose (*r* = −0.38), TG (*r* = −0.48), and HDL-C (*r* = 0.52) levels.

## 3. Discussion

We have shown that SAT biomarkers and architecture differ markedly in subjects with MetS compared to matched controls [31]. Specifically, we have documented that patients with nascent MetS (without the confounding conditions of diabetes and/or CVD) have increased levels of adipokines and decreased levels of adiponectin that are related to insulin resistance and inflammation. Furthermore, we have shown that this dysregulation of adipokines is not accounted for simply by increased adiposity, suggesting that other aspects of MetS contribute to both the proinflammatory and insulin resistant states of the syndrome. Also MetS should be classified as a high-risk obesity state based on our findings. Furthermore, at least 20 percent of our cohort were not obese, underscoring the high risk of metabolic syndrome in both obese and nonobese individuals.

Furthermore, we have documented a significant increase in macrophages in SAT and abundant CLS which appear to surround a hypoxic environment triggered by adipocyte death in subjects with MetS [33–35]. For unclear reasons, there were no significant correlations between CLS and biomarkers of inflammation in our studies; however, one can speculate that the CLS could be predominantly of the M2 macrophage phenotype participating in tissue remodeling. Also, we have shown that the chemokine MCP-1 is increased in SAT from MetS subjects, and, since this chemokine facilitates the homing of macrophages to such tissue depots, our data suggest that the SAT may indeed be a key player in MetS and its associated comorbidities. Moreover, because SAT is easy to access and SAT biopsies can be performed in large-scale clinical studies, and since the expression of inflammatory genes in SAT compares well with VAT [25, 26], the evaluation of SAT in subjects with and without MetS may provide novel insights into the syndrome's pathogenesis and serious sequelae.

Our data are also consistent with some but not all studies that have reported mRNA/gene expression profiles in SAT in subjects with MetS [36, 37]. Specifically, Gormez et al. reported increased mRNA levels of TNF-*α* and leptin but not adiponectin in SAT from subjects with MetS versus controls [36], whereas our data showed only increased amounts of SAT-secreted and circulating leptin concentrations. However, Gormez et al. studied MetS patients with CAD with 88% of the subjects having concomitant diabetes and dyslipidemia. Thus, one cannot appropriately ascribe their findings to MetS alone, and the additional confounding effects of medical conditions and medications cannot be excluded. Furthermore, Sacks et al. [37] reported no changes with respect to IL-1*β* gene expression levels in SAT from 15 subjects with MetS with CAD versus controls, whereas our data (using a much larger sample size) showed an increased amount of IL-1*β* released from SAT and in plasma. However, unlike in our studies, Sacks et al. [37] focused on a few selective biomediators/biomarkers with the confounding of CAD. The reported differences could also be the result of posttranscriptional processes, and thus highlight the problem with inferring that gene expression directly correlates with protein expression, the focus of our studies since it denotes function.

The novelty of our study is that we evaluated subjects with nascent MetS (without the confounding of diabetes and/or CVD) and assayed both plasma and SAT-secreted levels of adipokines. It is imperative to study MetS at this nascent stage in order to obtain a better understanding of the pathophysiology of this common disorder. Thus, our data suggest that these circulating adipokines function as biomediators that contribute to the increased risk for both diabetes and CVD in MetS subjects [38–40].

It is also important to note that CRP levels are significantly increased in the SAT of MetS subjects compared to controls. Although CRP appears to be predominantly produced in the liver, previous investigators have shown that there is increased CRP gene expression from adipose tissue [41] and vascular endothelium [42], a component of the stromal vascular fraction. These data thus support the notion that MetS is a proinflammatory state and that the adipose tissue of MetS contributes to the increased inflammation of these subjects.

The essential role of SAT in metabolic homeostasis has best been described in lipodystrophic syndromes, where its absence leads to ectopic fat accumulation in the liver and skeletal muscle with concomitant insulin resistance [43]. Although a deficiency in SAT has been previously associated with MetS, our data demonstrate that the SAT may also be a key player in the pathogenesis of MetS, consistent with findings from the Framingham Heart Study showing that larger volumes of SAT were associated with more cardiometabolic risk factors [22].

Another important observation from our studies is that MCP-1 appears to correlate well with insulin resistance and inflammation. Previous studies in rodents have shown that knockout of the MCP-1 receptor results in decreased hepatic inflammation and steatosis [44] and decreased adipose tissue macrophages [45], underscoring the importance of SAT MCP-1 in MetS subjects. The exact mechanisms by which MCP-1 contributes to both insulin resistance and increased inflammation in MetS need to be elucidated in future studies.

Moreover, we have made the novel observation that the SAT in subjects with nascent MetS (uncomplicated by the comorbidities of diabetes and/or CVD) has increased macrophage recruitment with cardinal CLS in greater abundance. Furthermore, these cells in SAT conspire to produce increased levels of biomarkers that correlate with both insulin resistance and low-grade inflammation, potentially presaging the subsequent increased risk for diabetes and CVD. 

In addition, we have recently demonstrated abnormal circulating and SAT-secreted chemerin and omentin-1 levels in subjects with nascent MetS [17]. Chemerin is a novel adipokine that is produced by both adipose tissue and liver; moreover, it is a chemoattractant for immune cells such as macrophages and promotes adipocyte differentiation [46]. Chemerin levels have also been shown to be higher in obesity, some features of MetS, diabetes, and nonalcoholic fatty liver disease [46–48], and it appears to induce insulin resistance in skeletal muscle, the major site of peripheral insulin resistance [49]. In our studies, we have made the novel observation that both plasma and SAT levels of chemerin are higher in subjects with nascent MetS, suggesting that chemerin could be involved early in the pathogenesis of the syndrome. Previously, in Caucasian subjects with Mets (including some with concomitant diabetes), serum chemerin levels were reported to be significantly increased; however, they were not adjusted for adiposity [50]. However, the investigators did not find a correlation between insulin resistance, obtained by two measures (HOMA and the quantitative insulin sensitivity check index (QUICKI)) and chemerin concentrations [50]. In a subsequent study in Korean subjects [51], the authors suggested that the ratio of chemerin to adiponectin might be a good predictor of MetS but did not report on adiposity-adjusted differences between patients with MetS and controls. Also, Dong et al. reported increased chemerin levels in patients with MetS (41% on statin therapy) with and without CAD and suggested it was an independent predictor of angiographic CAD [52]. However, they too did not correct for adiposity compared to controls, and thus we are unclear if this is a manifestation of MetS per se. Nonetheless, these studies collectively suggest a role for chemerin in the pathogenesis of MetS and its use as a biomarker to predict the syndrome needs to be urgently elucidated. 

Furthermore, based on our investigations, it appears that higher plasma chemerin levels in subjects with MetS emanate largely from the adipose tissue; however, we cannot exclude the contribution of other sources of chemerin production such as the liver. But, since VAT is not a major source of chemerin [53], our studies highlight the contribution of SAT to circulating chemerin levels and its use as a potential biomarker of SAT dysregulation. Moreover, our findings demonstrate higher SAT and plasma chemerin concentrations independent of obesity in nascent MetS, and also confirm significant correlations with insulin resistance, inflammation, BP, and dyslipidemia in nascent MetS, suggesting a potential role of chemerin in MetS and its sequelae [46–49]. 

As opposed to chemerin, omentin is predominantly expressed and secreted by VAT [54, 55] and appears to have insulin-sensitizing actions [55]. Furthermore, its levels are lower with both obesity and diabetes [55, 56]. We have documented lower levels of omentin-1 in nascent MetS in both SAT and plasma; moreover, lower omentin-1 levels persisted following correction for obesity in both plasma and in SAT [13]. Shang et al. have reported lower serum omentin-1 levels in patients with MetS (23% on statins and 32% on angiotensin-converting enzyme inhibitors/angiotensin receptor blockers) [57]; however, they did not correct for the increased BMI and waist circumference. Thus, they were unable to conclude that this correlation is a feature of MetS per se. Our study therefore adds to the published literature by documenting lower omentin release from SAT in subjects with nascent MetS independent of obesity. Moreover, since adipose tissue is the major source of omentin, we suggest that the lower secretion of omentin from SAT in subjects with nascent MetS establishes the presence of omentin deficiency in the syndrome as well. 

**Figure 1:**
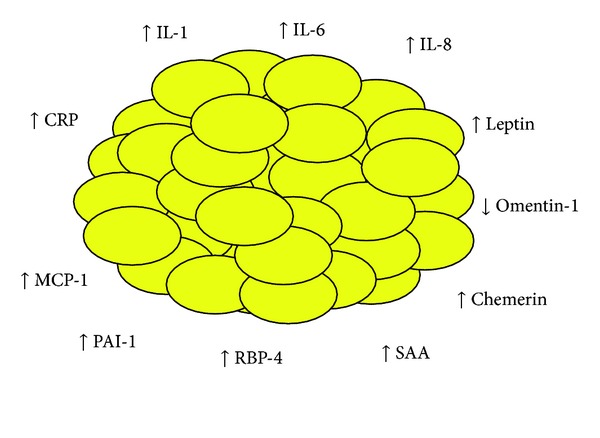
Schema depicting adipokine dysfunction in metabolic syndrome. Abbreviations: MCP-1, monocyte chemotactic protein-1; RBP-4, retinol-binding protein-4; SAA, serum amyloid A; CRP, C-reactive protein; IL, interleukin; PAI-1, plasminogen activator inhibitor-1.

In our studies, omentin levels significantly correlated with glucose (*r* = −0.43), TG (*r* = −0.50), and HDL-C (*r* = 0.53) concentrations, all features of MetS, but not with CRP levels and HOMA. Furthermore, although we showed higher plasma resistin concentrations in the subjects with MetS (that corrected with adiposity) versus the controls, we did not demonstrate higher SAT resistin levels. Thus, we can conclude that this cytokine, which in humans arises mainly from activated leukocytes [58], is a marker for the higher leuckoyte activity that we reported previously [59, 60]. Moreover, since visfatin levels were not different in MetS versus control subjects, we cannot confirm a role for visfatin in the etiology of MetS. A schematic representation of the induced SAT dysregulation in subjects with nascent MetS is depicted in Figure 1. 

## 4. Conclusions

We have made the novel observations that (i) the SAT in subjects with nascent MetS has increased macrophage recruitment with CLS in greater abundance; (ii) the SAT in subjects with nascent MetS produces increased levels of biomarkers that correlate with both insulin resistance and low-grade inflammation, potentially presaging the subsequent increased risk for diabetes and CVD; and (iii) there is a dysregulation in both SAT-derived chemerin and omentin-1 in subjects with nascent MetS, suggesting a possible role of these adipokines in both diabetes and CVD. Future investigations should study different fat depots to determine if our findings in a predominantly Caucasian population on SAT biology dysregulation are relevant to different race groups. Finally, comparing different fat depots in the same population with nascent metabolic syndrome will help decipher their role in adipokine dysregulation, insulin resistance, and inflammation in the pathobiology of this galloping epidemic.

## Figures and Tables

**Table 1:** Subcutaneous adipose tissue biomarker levels.

	Controls	MetS
Adiponectin (ng/g)	4.2 (1.3, 5.6)	3.7 (1.2, 4.6)
Leptin (ng/g)	3.0 (2.1, 6.2)	7.3 (3.8, 18.6)*
RBP-4 (ng/g)	11.1 (6.4, 18.4)	29.1 (16.2, 33.7)**
CRP (ng/g)	2.5 (2.3, 7.9)	5.4 (3.4, 19.1)*
SAA (ng/g)	14.8 (5.1, 34.2)	25.3 (14.5, 55.7)*
PAI-1 (ng/g)	3.2 (2.2, 6.5)	5.6 (3.1, 9.9)**
MCP-1 (ng/g)	6.7 (4.3, 9.1)	22.1 (11.8, 33.5)**
IL-1*β* (ng/mg protein)	31.1 (21.2, 45.1)	39.7 (24.8, 61.5)*
TNF (ng/mg protein)	3.7 (1.9, 4.6)	3.8 (2.9, 5.3)
IL-6 (ng/mg protein)	16.5 (10.6, 24.5)	18.7 (12.7, 33.2)*
IL-8 (ng/mg protein)	10.9 (5.1, 14.2)	17.4 (14.5, 27.3)**

*Copyright 2011, The Endocrine Society. Reproduced with permission from the Endocrine Society.*

Data are expressed as median (25th percentile and 75th percentile).

*P < 0.05, **P < 0.001 compared to controls.

**Table 2:** Novel adipokine concentrations.

Variable	Controls (*n* = 30)	MetS (*n* = 45)	*P* value MetS versus control
hsCRP (mg/L)	1.3 (0.5, 4.0)	3.1 (1.6, 5.4)	0.006
HOMA-IR	1.1 (1.0, 2.8)	2.8 (1.9, 5.1)	0.0001
Plasma chemerin (ng/mL)	271 ± 53	366 ± 64	<0.0001
	*n* = 20	*n* = 37	*(0.0005)
SAT chemerin (ng/mg protein)	3.05 ± 0.94	3.94 ± 0.74	0.001
	*n* = 30	*n* = 45	
Plasma omentin (ng/mL)	27 ± 14	16 ± 5	0.004
	*n* = 16	*n* = 16	*(0.03)
SAT omentin (ng/mg protein)	0.31 ± 0.09	0.22 ± 0.10	0.01
	*n* = 30	*n* = 45	
Plasma resistin (ng/mL)	1.8 (1.5, 2.5)	2.4 (1.7, 3.1)	0.04
	*n* = 21	*n* = 31	*(0.07)
SAT resistin (ng/mg protein)	0.16 ± 0.06	0.17 ± 0.05	NS
	*n* = 30	*n* = 45	
Plasma visfatin (ng/mL)	0.57 (0.38, 0.71)	0.59 (0.31, 0.96)	0.14
	*n* = 22	*n* = 36	*(0.13)
SAT visfatin (ng/mg protein)	0.17 ± 0.09	0.21 ± 0.1	NS
	*n* = 30	*n* = 45	

*(*P* value adjusted for age and BMI).

Results are presented as mean ± standard deviation or median (25th percentile and 75th percentile).
